# Chronic Administration of Recombinant Human Erythropoietin Induces Angiogenesis in Healthy Mouse Brain

**DOI:** 10.7759/cureus.68362

**Published:** 2024-09-01

**Authors:** Olga Pagonopoulou, Vasiliki Papadatou, Stylianos Tologkos, Anna Efthimiadou, Lambropoulou Maria

**Affiliations:** 1 Neurophysiology, Democritus University of Thrace, Alexandroupolis, GRC; 2 Histology-Embryology, Democritus University of Thrace, Alexandroupolis, GRC; 3 Physiology, Independent Researcher, Alexandroupolis, GRC

**Keywords:** immunohistochemistry staining, histology, angiogenesis, healthy mouse brain, erythropoietin

## Abstract

Introduction

The hematopoietic growth factor erythropoietin (EPO) plays an important role in apoptosis and oxidative stress attenuation as well as the promotion of angiogenesis in several tissues. Systemically administered EPO has beneficial effects on rabbits subjected to subarachnoid hemorrhage or stroke. So far, the angiogenic effect of EPO has been documented after an experimentally induced stroke or subarachnoid hemorrhage. In our study, we examined the possible angiogenic effect of chronic treatment with recombinant human erythropoietin (rHuEPO) under normal conditions, in an attempt to clarify if the existence of a lesion or oxygen deprivation is necessary to initiate the angiogenic effect of EPO.

Materials & methods

BALB/c mice were used and were divided into three groups as follows: group A (no treatment), group B (saline only), and group C (7000 U rHuEPO per week for three weeks by intraperitoneal injection). The number of CD31- and CD34-positive endothelial cells was assessed in mouse brain preparations under control conditions and after treatment with rHuEPO.

Results

There was no difference between the mean numbers of CD31 and CD34 cells among the different groups. The mean number of vessels in group A and group B was almost the same (18 ± 2 vessels per optical field). However, the number of brain vessels in group C (EPO treatment) increased significantly by 44% compared to controls (26 ± 4 vessels per optical field, P < 0.05).

Conclusion

These data indicate that no lesion or oxygen deprivation is needed to initiate the angiogenic effect of EPO in healthy mouse brains.

## Introduction

Erythropoietin (EPO), a low molecular weight glycoprotein, was the first cloned hematopoietic growth factor, and its single physiological effect was originally considered to be erythropoiesis [1]. However, this premise was later changed because EPO and its target receptor were found to be expressed in organs other than the liver and kidneys, such as the heart, uterus, bone marrow macrophages, neurons, astrocytes, brain endothelial cells, microglia, and oligodendrocytes [2]. Its original action as a hemopoietic cytokine is to stimulate the proliferation and differentiation of erythroid progenitor cells. In the fetal environment, EPO is produced by the liver hepatocytes, while in adults, EPO production is taken on by the peritubular fibroblasts of the kidney [3,4].

Therefore, although the specific functions of EPO and its receptor in these sites are not yet completely clarified, there is accumulating evidence suggesting a wider biological role of EPO/EPO receptors not related to erythropoiesis. EPO has been suggested to have a neuroprotective action through inhibition of apoptosis of neuronal cells, an antioxidant effect via inhibition of formation of reactive oxygen species, and has also been shown to stimulate angiogenesis [4-7].

Angiogenesis takes place in various physiological and pathological conditions such as embryonic development (related to vasculogenesis, i.e., the formation of capillaries from endothelial cells differentiating in situ from groups of mesodermal cells), wound healing, the menstrual cycle, chronic inflammations, and tumors. Indications of the angiogenic role of EPO have been shown so far in several tissues such as the kidney (where chronic treatment with EPO induces an increase in the number of vessels), the heart (where chronic treatment with EPO induces an increase in the number of vessels), and the uterus (where injection of EPO into the uterine cavity of ovariectomized mice led to blood vessel formation in the endometrium) [8-10].

In the brain environment, the role of angiogenesis may be even greater as it may provide indirect neuroprotection, which is closely related to neuronal survival in patients with ischemic stroke. This beneficial action of angiogenesis may result from the restoration of blood flow in the ischemic border through arteriolar growth and capillary formation during ischemia [8].

Systemically administered EPO has been shown to exert beneficial effects on rabbits subjected to subarachnoid hemorrhage [11] and stroke [12], whereas intranasal administration of recombinant human erythropoietin (rHuEPO) protects rats against focal cerebral ischemia [13]. EPO’s action in the brain has also been investigated in rat models of cardiac ischemia/reperfusion injury [14], showing neuroprotective, anti-apoptotic, and anti-inflammatory effects.

So far, all studies concerning the angiogenic effect of EPO were performed after an experimentally induced stroke or subarachnoid hemorrhage. In our study, we examined the possible angiogenic effects of chronic treatment with EPO under normal conditions to clarify if the presence of a lesion or if oxygen deprivation conditions are necessary to initiate the angiogenic effects of EPO, or if EPO administration could exert the same angiogenic effect in normal intact brain tissue.

Our objective was to evaluate the possible angiogenic effects of chronic treatment with EPO in healthy brain tissue to assess if a lesion or oxygen deprivation condition is needed to initiate the angiogenic effects of EPO.

This article was previously presented as a meeting abstract at the 21st Annual Conference of the Hellenic Society for Neuroscience, November 30th - December 1st, 2007, Thessaloniki, p 170.

## Materials and methods

Animal model

In this study, we used three-month-old BALB/c mice weighing 20-25 g. The animals were housed in separate cages with free access to water and laboratory chow under controlled conditions of light and temperature. They were divided into three groups of 10 mice each. Group A received no treatment, group B received saline only, and group C received 7000 U rHuEPO per week for three weeks administered by intraperitoneal (i.p.) injection. The dose and route of administration of rHuEPO were chosen based on previous reports [15,16]. The mice were sacrificed 21 days after the onset of treatment. On that day, mice were decapitated, the brains were removed, and then prepared for histological examination. Animal care and handling was carried out based on the Directive 86/609/EEC guidelines.

Drugs

Recombinant erythropoietin was purchased from Janssen-Cilag Ltd, Saunderton, UK.

Hematocrit measurement

A blood sample for hematocrit determination was drawn via the tail vein before and weekly until the end of rHuEPO treatment. Hematocrit measurement was performed using a microcentrifuge.

Cerebrospinal fluid EPO concentration

CSF samples were collected immediately and 30 minutes after EPO administration from the cisterna magna. EPO concentrations were determined using enzyme-linked immunosorbent assay (ELISA) following the manufacturer’s instructions (RE5604, Immuno-Biological Laboratories, Hamburg, Germany). The lower detection limit was 2 mU/ml.

Histological examination

Tissue specimens were fixed in formalin and embedded in paraffin according to standard procedures. Sagittal sections (2 μm thick) of mouse brains were cut and posed on microscope slides. Slides were stained with conventional hematoxylin and eosin (H&E). Unstained slides were obtained for the detection of CD31 and CD34 via immunohistochemistry. We used a CD31 monoclonal mouse anti-human antibody (CD31, clone: INN-PECAM-1; Novocastra, Newcastle Upon Tyne, UK) and CD34 lyophilized monoclonal antibody (clone: QBEnd/10; Novocastra), which recognize the surface antigens CD31 and CD34, respectively, of endothelial cells.

Immunohistochemistry

Immunohistochemistry was performed with CD31 (Novocastra) and CD34 (Novocastra) on serial sections. Sections (4 µm) of representative blocks from each case were deparaffinized, dehydrated, and treated with 0.3% H2O2 for five minutes in methanol to prevent endogenous peroxidase activity. Slides were then incubated overnight with CD31 and CD34 at 1:50 and 1:80 dilutions, respectively. Control slides were incubated for the same period with normal serum (negative control). Positive controls were always run in the assay. We used the Envision Kit (Dako, Glostrup, Denmark) according to the manufacturer’s instructions except that the signal amplification was performed with 3-amino-9-ethylcarbazole (AEC) (AEC + substrate chromogen, ready-to-use, Agilent DAKO, K3461). Sections were then briefly counterstained with Mayer’s hematoxylin, mounted, and examined under a Nikon ECLIPSE 50i light microscope (Nikon Corporation, Tokyo, Japan). High visualization fields (hot spots) were selected and then 10 optical fields with a ×200 magnification were randomly chosen within each hot spot and each endothelial cell or cell cluster that showed antibody staining. The average of measurements of 10 fields with increased vascularization was considered as the final microvessel density.

Statistical analysis

The number of vessels per optical field was analyzed in every specimen and data are presented as the mean ± standard error of the mean (SEM). Descriptive statistics were calculated for all groups. Comparisons among experimental groups were performed using one-way ANOVA, followed by Tukey’s honest significant difference (HSD) post hoc test (SPSS, version 28; IBM Corp., Armonk, NY).

## Results

Cerebrospinal fluid EPO concentration

The administration of 7000 U/kg rHuEPO i.p. resulted in an increase of about 110 mU/ml in EPO concentration in the CSF within 30 minutes from the injection. These data confirm previous reports suggesting that systemically administered EPO can cross the blood-brain barrier [17,18].

Immunohistochemistry

The number of CD31- and CD34-positive endothelial cells was assessed in mouse brain preparations under control conditions and after treatment with rHuEPO. There was no difference between the numbers of CD31 and CD34 cells among the different groups. The results of our experimental study are presented in Table 1.

**Table 1:** Mean value (±SEM) of the number of vessels in EPO-treated and non-treated animals in CD31- and CD34-stained cells. *: p < 0.01; SEM: standard error of the mean; EPO: erythropoietin; rHuEPO: recombinant human erythropoietin.

	No. of vessels (CD31)	No. of vessels (CD34)
Not treated	19 ± 2	18 ± 2
Saline treated	17 ± 2	19 ± 2
rHuEPO treated	26 ± 3*	27 ± 4*

**Figure 1:**
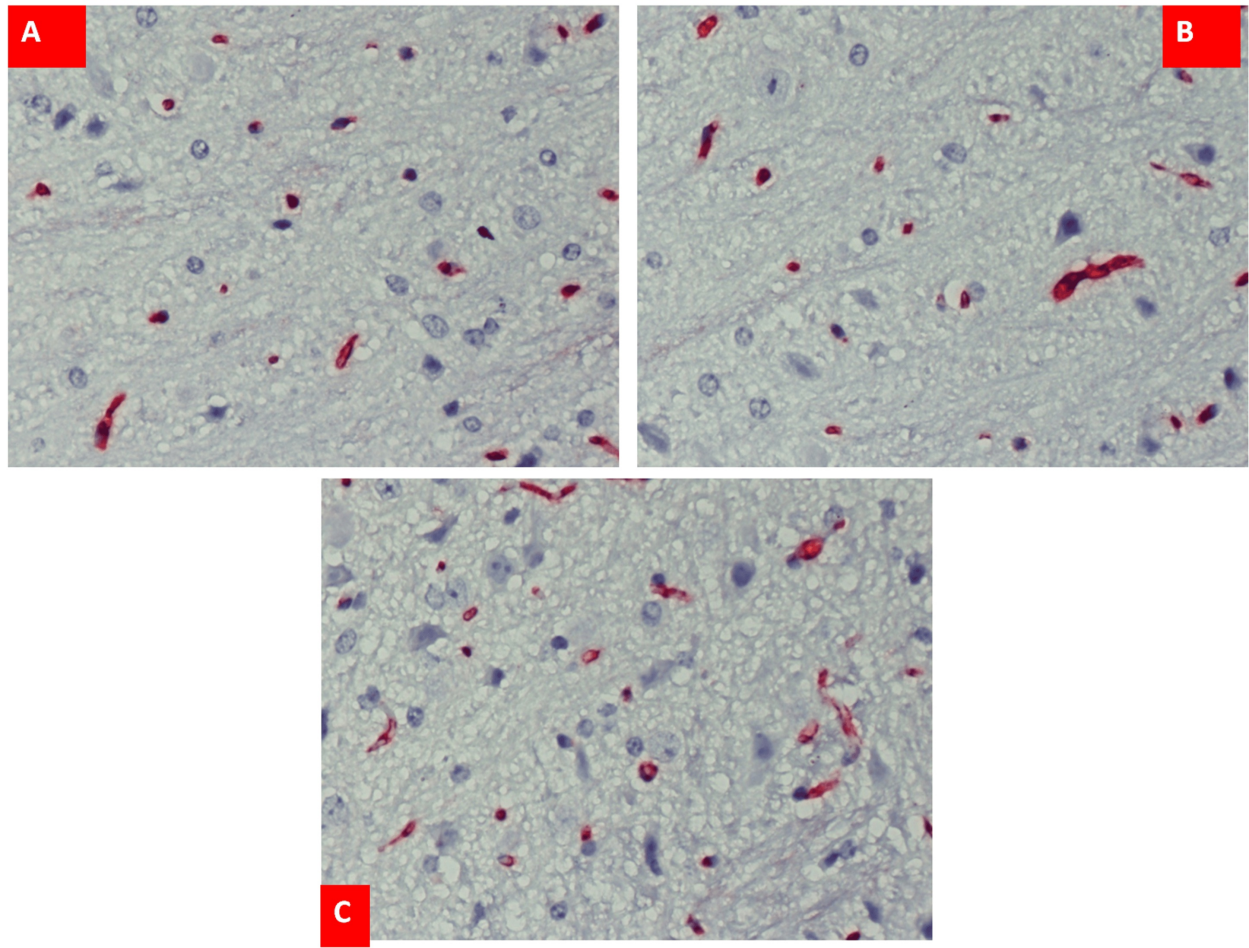
CD31 immunohistochemistry results for (A) group A, (B) group B, and (C) group C. Increased number of blood vessels in group C compared to the other groups (x200 magnification).

Group A mice received no treatment (Figure 1A). Saline-treated mice are indicated as the control group (Figure 1B). There was no statistically significant difference in the mean number of vessels between the non-treated and saline-treated mice (groups A and B, respectively). However, the number of brain vessels in group C (rHuEPO-treated mice) was increased with statistical significance by 44% compared to controls (P < 0.05; Figure 1C).

## Discussion

The restoration of blood supply in the locus of an ischemic tissue plays a critical role in tissue repair and functional recovery. Studies using several experimental models of brain injury have shown that systemic EPO administration has beneficial effects on vascular endothelial cell survival and promotes angiogenesis, which helps in cerebral blood flow restoration after permanent focal cerebral ischemia in mice [19]. Another study on rats has also shown that chronic administration of EPO enhances neurogenesis and angiogenesis and improves neurological function in rats in vivo after an embolic stroke [12]. These beneficial effects of EPO are of interest concerning the potential therapy or recovery after an ischemic stroke [19]. In vitro experiments have also shown that rHuEPO has angiogenic effects on mouse brain endothelial cells and this increase may be induced via an increase in vascular endothelial growth factor (VEGF) levels [12]. So far, all angiogenic effects of EPO are induced after an ischemic stroke. However, given that EPO is used as a therapeutic agent in several cases with adverse effects [20,21] and also because EPO administered systemically can cross the blood-brain barrier [17,22,23], we wanted to clarify if EPO administration has an angiogenic effect in healthy brain tissue. In our study, we show for the first time that, regardless of a pre-existing stroke or ischemic condition, chronic administration of a high dose of rHuEPO could lead to statistically significant increases in the number of blood vessels in the mouse brain. This has also been proven for other tissues such as the mouse heart [10] and the rat kidney [9].

Many clinical trials have evaluated the beneficial effects of EPO for treating anemia associated with renal failure. It has been proven to enhance the production of erythroblasts, thereby alleviating the symptoms of anemia in patients suffering from chronic kidney disease. This plays a crucial role in the management of these patients and can vastly increase their quality of life. In addition to renal failure, EPO has also been investigated for its potential to treat anemia associated with cancer. Cancer-related anemia can result either from the disease itself or as a side effect of chemotherapy. By increasing red blood cell count, EPO can help improve oxygen delivery to tissues. However, two other studies have shown that patients treated with EPO can have worse outcomes than their placebo-treated counterparts [24,25]. These studies raised concerns about the safety and efficacy of EPO in certain patient populations. Specifically, it was observed that in some cases, the use of EPO can lead to severe side effects, including thromboembolic events and increased mortality. These results suggest that while EPO can be beneficial for some patients, it may pose a risk to others, thus highlighting the need for careful use on a case-by-case basis. Furthermore, EPO seems to have a mitogenic and an anti-apoptotic effect on various types of cells [26-28]. The mitogenic effect refers to EPO's ability to stimulate cell division and proliferation, which can be beneficial in tissues where cell regeneration is needed. The anti-apoptotic effect means that EPO can help protect cells from various forms of stress or injury.

However, Um et al. (2007) showed that EPO has this antiapoptotic effect on cancer cells as well [29]. This finding is concerning because while the anti-apoptotic properties of EPO can be protective for normal tissues, they could potentially be harmful in patients with cancer. By preventing the apoptosis of cancer cells, EPO might inadvertently help and promote the survival and proliferation of malignant cells. This dual effect of EPO highlights both the complexity of its role in different biological settings and the importance of further research to fully understand the implications of its use. Liu et al. (2020), in their recent study, support that in lung cancer models, EPO plays a strong role in angiogenesis as well besides its well-established role in erythropoiesis by participating in new blood vessel formation, thus raising the risk for thrombosis and metastasis in vivo [30].

A limitation of this study is that we only used BALB/c mice. Extending the study to different strains and species would help validate the findings. Secondly, the size group is relatively small, so a larger cohort of mice would provide more robust data and validate the results. Further research exploring the underlying molecular mechanisms by which EPO induces angiogenesis in the absence of injury or hypoxia would enhance the understanding of EPO's angiogenic effects.

## Conclusions

In this study, we proved that a high dose of rHuEPO can induce an increase in blood vessels in the brain tissue of mice. We elucidated for the first time in an animal study that a lesion or an oxygen deprivation event is not necessary for the angiogenic effect of rHuEPO. Recent studies have also reported on the angiogenic effect of rHuEPO. These data combined with our findings about the angiogenic effect of systemically administered rHuEPO in the healthy brain tissue indicate that special care should be taken when EPO is administered systemically for therapeutic reasons.
